# Do I have an ethical dilemma?

**DOI:** 10.4103/0974-620X.64226

**Published:** 2010

**Authors:** Alex V. Levin

**Affiliations:** Pediatric Ophthalmology and Ocular Genetics, Wills Eye Institute, Thomas Jefferson University, Philadelphia, Pennsylvania, USA

Have you ever had that gnawing feeling in the pit of your belly that something is wrong? Perhaps when a diagnosis just doesn’t seem to fit or something isn’t going smoothly during surgery? The same feeling may arise when ethical dilemmas occur. Perhaps it was when a patient asked you whether you would be doing the surgery when you knew it was a case that you would likely let a resident perform. Or maybe you made an error that left you debating whether to inform the patient. Perhaps you felt uncomfortable when a drug salesman asked you out to dinner or when you thought about how much you should tell a patient regarding the risks of surgery.

That feeling that gives us pause and discomfort is often referred to as “moral distress”. It is in these situations that we should raise our internal attention to identify and address the dilemma. The first step is the recognition that one is in a moral quandary. Dissecting the issue further might help to identify paths for resolution. To do so, one might use different lenses to examine the situation: ethics, law, policy, empiric research, philosophy, and moral foundations. These spheres overlap like a ven diagram, sometimes being exclusive and other times sharing commonalities. By considering the features of each of these areas, we might gain useful perspective on handling our ethical dilemma in real time.

Many authors have tried for many decades to define the word ethics. But its plurality is likely most important. Each of us comes to a dilemma with our own background in terms of our upbringing, values, religion, experiences, life situations, and other socioeconomic and demographic factors. As we try to resolve our moral distress, we call upon our unique histories to give us a perspective that allows us to make our own “right decision” that fits our own “ethic”. Although it may be fair to say there is no right or wrong ethic, and it is important to recognize that our individual ethics, must also interact with the ethics of our colleagues, the patient, and the patient’s family. Doing “the right thing” becomes a meshwork of societal, professional, personal, and patient ethics. This may seem like a multidirectional wind in a storm, but we can use other lenses to help us find a compass to direct us appropriately.

Laws are imposed by society, usually in response to circumstances or events that society feels require regulation. Laws are generated either in the courtroom in the form of decisions which may or may not uphold previous precedents and also *de novo*, when the governmental legislature chooses to enact new laws. Failure to comply with the law elicits a punishment response from society which should be proportionate to the malfeasance. All of us are law breakers. Have you ever driven over the speed limit? Although the punishment for such a “crime” may simply be the annoyance and cost of a speeding ticket, more serious breaches of law can result in imprisonment or hefty fines. Laws are designed to consider society’s needs over individual desires. But law also protects the rights given to individuals from the actions of other individuals or organizations.

Policies are set by organizations such as hospitals and professional bodies. Although they do not rise to the level of law in terms of societal impact or punishment, they are created by these organizations to create internal standards of behavior for the purpose of quality management and the fulfillment of common objectives. Failure to adhere to policy can result in punishment which, is enacted by the organization rather than society. Punishment may range from a simple reprimand to the loss of one’s job. Policies are usually generated by committees and many times sit in binders on dusty shelves until one needs to call upon them to resolve a dispute or a situation of “misdoing”. Although policies, like law, may reflect societal or organizational ethics and values, there are more often called upon to resolve procedural quandaries rather than moral dilemmas.

An often overlooked resource for resolving ethical dilemmas is that of empiric research. Since the technology explosion of the 1960’s when we became able to save babies with increasing levels of prematurity and sustain life by mechanical means through technology such as artificial ventilation, thousands of studies have been conducted to examine the ethical implications of our actions. Both quantitative and qualitative research methods have been used to help enlighten physicians about resource allocation, disclosure of error, doctor’s involvement in medical industry, teaching ophthalmic surgery to trainees, patient preferences regarding pre-symptomatic testing for genetic disease, research ethics, and a whole host of ethics topics. This peer reviewed literature can be turned to for guidance just as we would turn to our medical journals for help in treating retinoblastoma, central retinal vein occlusion, or diabetic retinopathy. Ophthalmologists must be as familiar with this robust literature as they are with published works regarding procedures, medications, and disease.

Philosophy is an art and science which is rarely called upon by the ophthalmologist. Yet, deep thought has been given to many aspects of our professional lives in terms of philosophical schools such as deontology, utilitarianism, and others. The discourse of philosophers asks important questions about such topics as the potential for universal principles to guide our professional lives. How does one justify a principal such as “thou shall not kill” when we are faced with difficult decisions about end of life (or end of vision)? Are there times, when futility, should allow us to let an eye go blind?

Lastly, if not most importantly, we come to the lens of moral foundations. Medicine is based upon the doctor-patient relationship. This encounter is very distinct from the provider-consumer relationship in business transactions. Rather, the patient must entrust their physician, a situation whereby the patient becomes a supplicant asking their physician for help at a time when they are unable to help themselves and may even need to turn over some of their autonomy to the physician to allow for appropriate care making decisions. Entrustment is a very powerful aspect of this relationship, placing the physician in a powerful position that can easily be abused and sometimes is.

So when the ophthalmologist, has that “feeling in their gut,” that they are in an ethical dilemma, how does one use these lenses to help resolve the problem? As surgeons, sometimes we want a “quick fix” but that is not always easily available. One lens may prove to be more important in certain situations. For example, when debating whether to report a suspicion of child abuse, the law in many countries clearly states that the physician has a legal requirement to report to the appropriate authorities. This law trumps ethical concerns such as the patients’ privacy or the autonomy of the parents in their care for the child. If a doctor is debating on whether to inform the patient of a surgical complication, such as rupture of the posterior capsule during cataract extraction, she may be beholden to hospital policy (or in some jurisdictions even societal law) that mandates such disclosure even though the physician worries about the ethical implications of making a patient more frightful or confused. If a doctor worries about the ethical implications of his business relationships with medical industry, he can turn to numerous research studies that have clearly shown, that such relationships virtually always affect prescribing practices. But more often than not, the lenses are somewhat blurred and the view overlapping. In these situations, the ophthalmologists must rely on other means to find a resolution of the dilemma. Fortunately, several options exist.

Firstly, many hospitals now have ethics committees either in the form of committees which can deal with clinical dilemmas. Institutional review boards (also known as research ethics board) provide a means of insuring that both the researcher and the research subject are protected from harm, research that is conducted is of high quality, and resources are utilized appropriately. Some institutions have developed special pathways for approval of surgical innovations outside, the standard research stream. In the absence of clinical or research ethic committees, units and clinical services, can come together to discuss ethical dilemmas. Whether it be through classic “morbidity and mortality” conferences or ad hoc meetings of the clinical team, with or without invited guests such as clergy, lawyers, or even patients and their families, maybe useful when difficult situations arise. Even more informal is the appropriate inclusion of ethical concerns when teaching our trainees, doing bedside rounds, or scrubbing before our next case. These informal professional dialogues allow us to share our concerns, ethics, and perspectives in a way that helps us reflect on the troubling situations. Professional communication with our colleagues and coworkers is a critical step in helping to find our way through the moral uncertainties that are part of our everyday lives.

So when that uncomfortable feeling arises that tells us we have an ethical dilemma at hand, let us share that dilemma with others, talk and read, to help find our way. In doing so, we will make “right decisions” that benefit our patients and forward our profession. Making ethics part of our everyday lives through continuing education, teaching, research, and clinical practice will not only benefit our patients, but it will also enrich us as people and professionals.

